# Supplementary material for: RTEC-intrinsic IL-17–driven inflammatory circuit amplifies antibody-induced glomerulonephritis and is constrained by Regnase-1
Source: JCI Insight. 2021 Jul 8;6(13):e147505. doi: 10.1172/jci.insight.147505 (PMC8410033; doi:10.1172/jci.insight.147505)
Supplement: Supplemental data [file jciinsight-6-147505-s072.pdf]

Supplementary materials

Supplementary Figure Legends

Fig S1: Representative flow cytometry gating strategy for identification of $CD45^+$ subsets: Neutrophils ($CD45^+CD11b^+Ly6G^+$), macrophages ($CD45^+Ly6C^+Ly6G^-CD11b^{hi}F4/80^{hi}$), monocytes ($CD45^+Ly6C^+Ly6G^-CD11b^{low}F4/80^{low}$), B cells ($CD45^+B220^+$), $CD4^+$ T cells ($CD45^+CD3^+CD4^+$) and $CD8^+$ T cells ($CD45^+CD3^+CD8^+$) infiltration in the AGN kidneys were analyzed by flow cytometry on day 7 post-AGN induction. Representative dot plots showing gating strategy.

Fig S2: Reduced inflammatory cell infiltration in the nephritic kidney of RTEC-specific Il17ra deficient mice. *$Il17ra^{fl/fl}$* and *$Il17ra^{Cdh16}$* mice (*n*=3-7) were subjected to AGN. At day 7 post anti-GBM serum injection, **(A)** B, $CD4^+$ T and $CD8^+$ T cells numbers, and (**B**) neutrophils, macrophages, monocytes, B, $CD4^+$ T and $CD8^+$ T cells percentages from $CD45^+$ cells in the kidney were quantified by flow cytometry. The data is pooled from at least 2 independent experiments. Statistical analysis by Two-way ANOVA.

Fig S3: Increased inflammatory cell infiltration in the nephritic kidney of $Zc3h12a^{+/-}$ mice. *$Zc3h12a^{+/+}$* (WT) and *$Zc3h12a^{+/-}$* mice (n=3-6) were subjected to AGN. At day 7 p.i., **(A)** monocytes, **(B)** B, $CD4^+$ T, and $CD8^+$ T cell numbers, and (**C**) neutrophils, macrophages, monocytes, B, $CD4^+$ T and $CD8^+$ T cells percentages from $CD45^+$ cells in the kidney were quantified by flow cytometry. **(D)** Bone marrow (BM) cells from *$Zc3h12a^{+/-}$* and wild WT mice

were adoptively transferred into sub-lethally irradiated *Zc3h12a*$^{+/-}$ or WT recipients (n=5-8). Eight weeks later, successfully reconstituted mice were subjected to AGN and assessed for serum creatinine level. The data is pooled from at least 2 independent experiments. Statistical analysis by Two-way ANOVA (A-C) or One-way ANOVA (D).

Fig S4: Increased inflammatory cell infiltration in the nephritic kidney of RTEC-specific Regnase-1 deficient mice. *Zc3h12a*$^{fl/fl}$ and *Zc3h12a*^{Cdh16} mice (*n*=3-6) were subjected to AGN. At day 7 p.i., **(A)** CD45^{+} cell numbers and (**B**) neutrophils, macrophages, monocytes, B, CD4^{+} T and CD8^{+} T cells percentages from CD45^{+} cells in the kidney were quantified by flow cytometry. The data is pooled from at least 2 independent experiments. Statistical analysis by unpaired Student's t test (A) and Two-way ANOVA (B).

Fig S5: Deletion of ZC3H12A gene drives inflammatory mediator expression in the HK-2 cell line. HK-2 and HK-2$^{\Delta ZC3H12A}$ cells were stimulated with IL-17 and/or TNFα for 8 h. **(A)** Gene expression of *CEBPB* and *CEBPD* was measured by qPCR, normalized to *GAPDH*. **(B)** Cell lysates were evaluated for C/EBPβ and C/EBPδ protein expression by western blot. Protein relative abundance and representative image are shown. **(C)** *Lcn2* promoter activity was measured by luciferase assay in IL-17 Reporter HEK 293 cells after IL-17 stimulation. Data pooled from at least 3 independent experiments. Statistical analysis by Two-way ANOVA (A and B) and unpaired Student's t test (C).

Supplemental Figures

Figure S1

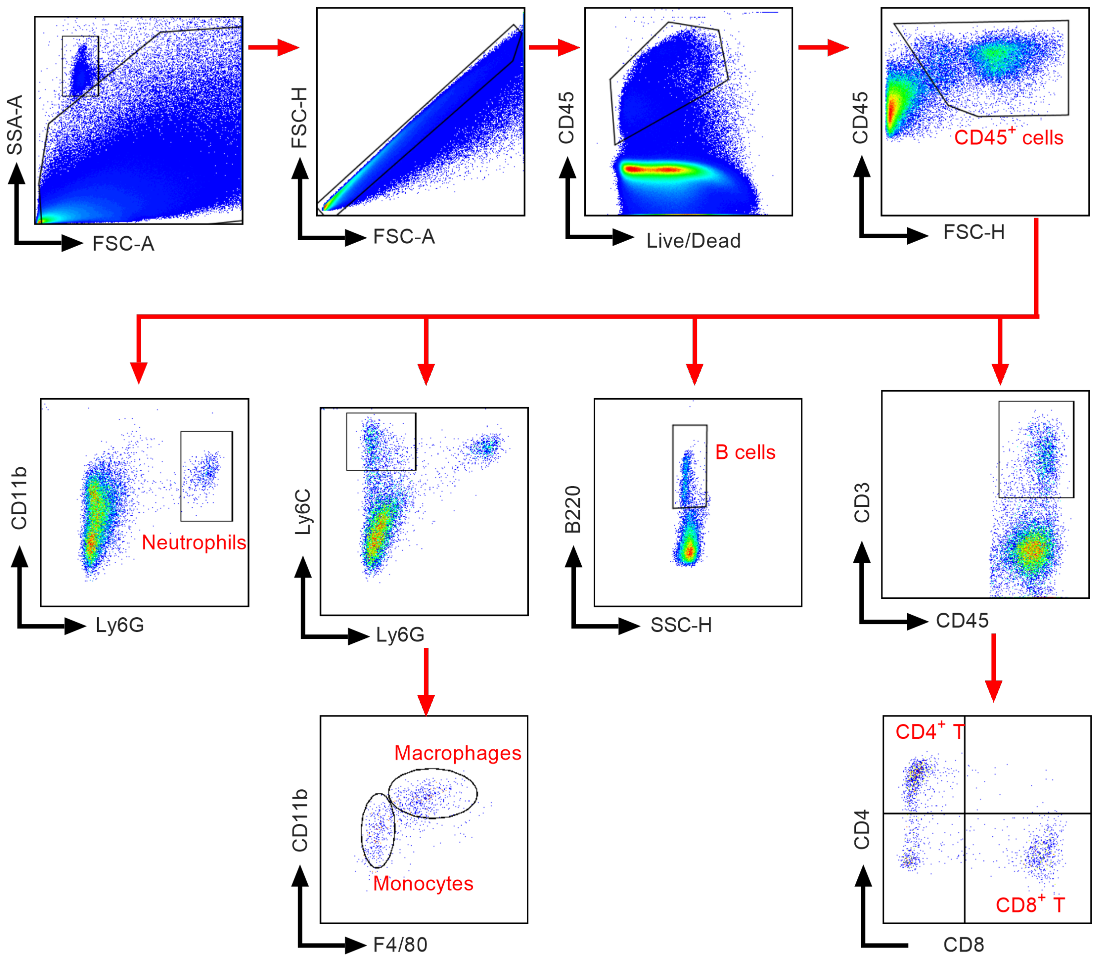

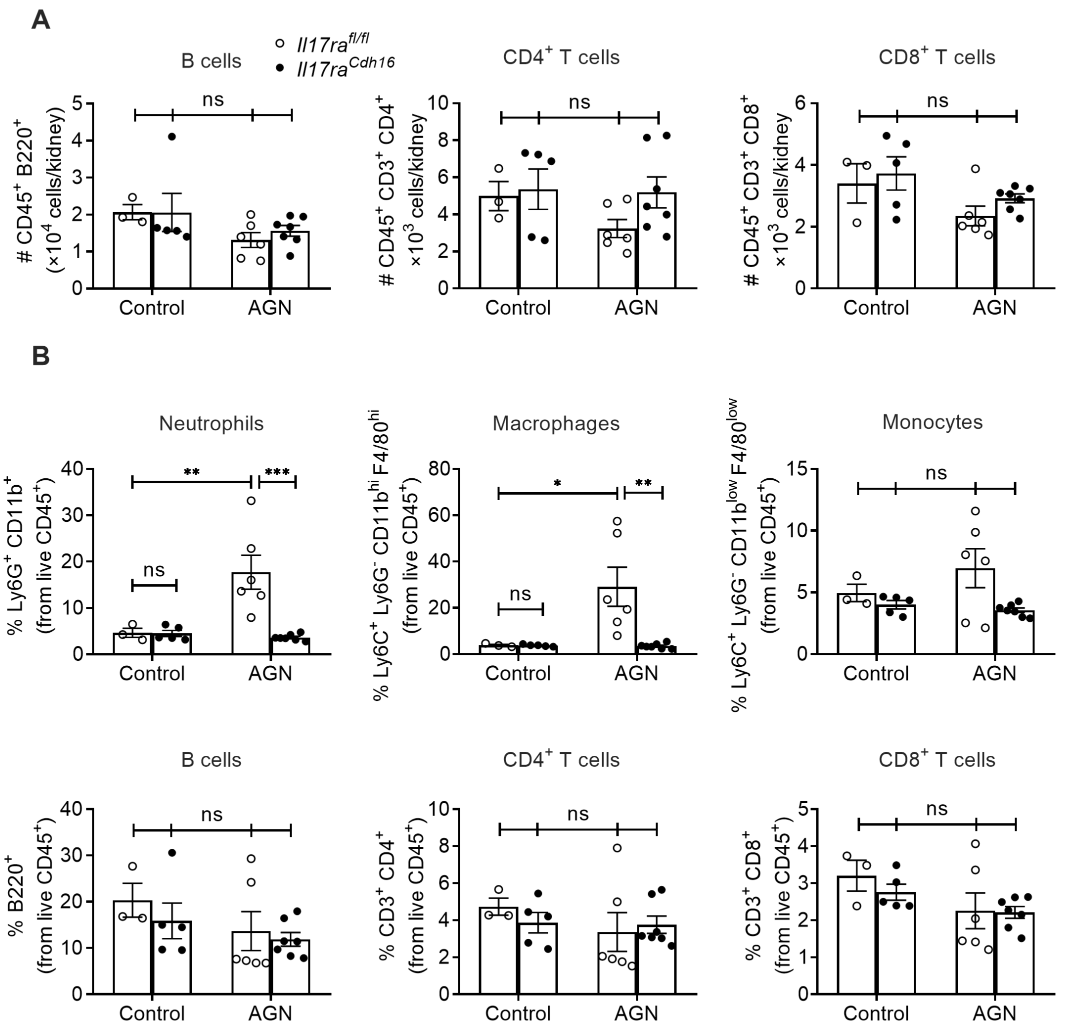
A
B cells
Il17ra fl/fl
Il17ra Cdh16
CD4+ T cells
CD8+ T cells
CD45+ B220+ (×10^4 cells/kidney)
CD45+ CD3+ CD4+ ×10^3 cells/kidney
CD45+ CD3+ CD8+ ×10^3 cells/kidney
ns
Control
AGN
B
Neutrophils
Macrophages
Monocytes
% Ly6G+ CD11b+ (from live CD45+)
% Ly6C+ Ly6G- CD11b hi F4/80 hi (from live CD45+)
% Ly6C+ Ly6G- CD11b low F4/80 low (from live CD45+)
B cells
CD4+ T cells
CD8+ T cells
% B220+ (from live CD45+)
% CD3+ CD4+ (from live CD45+)
% CD3+ CD8+ (from live CD45+)

Figure S3

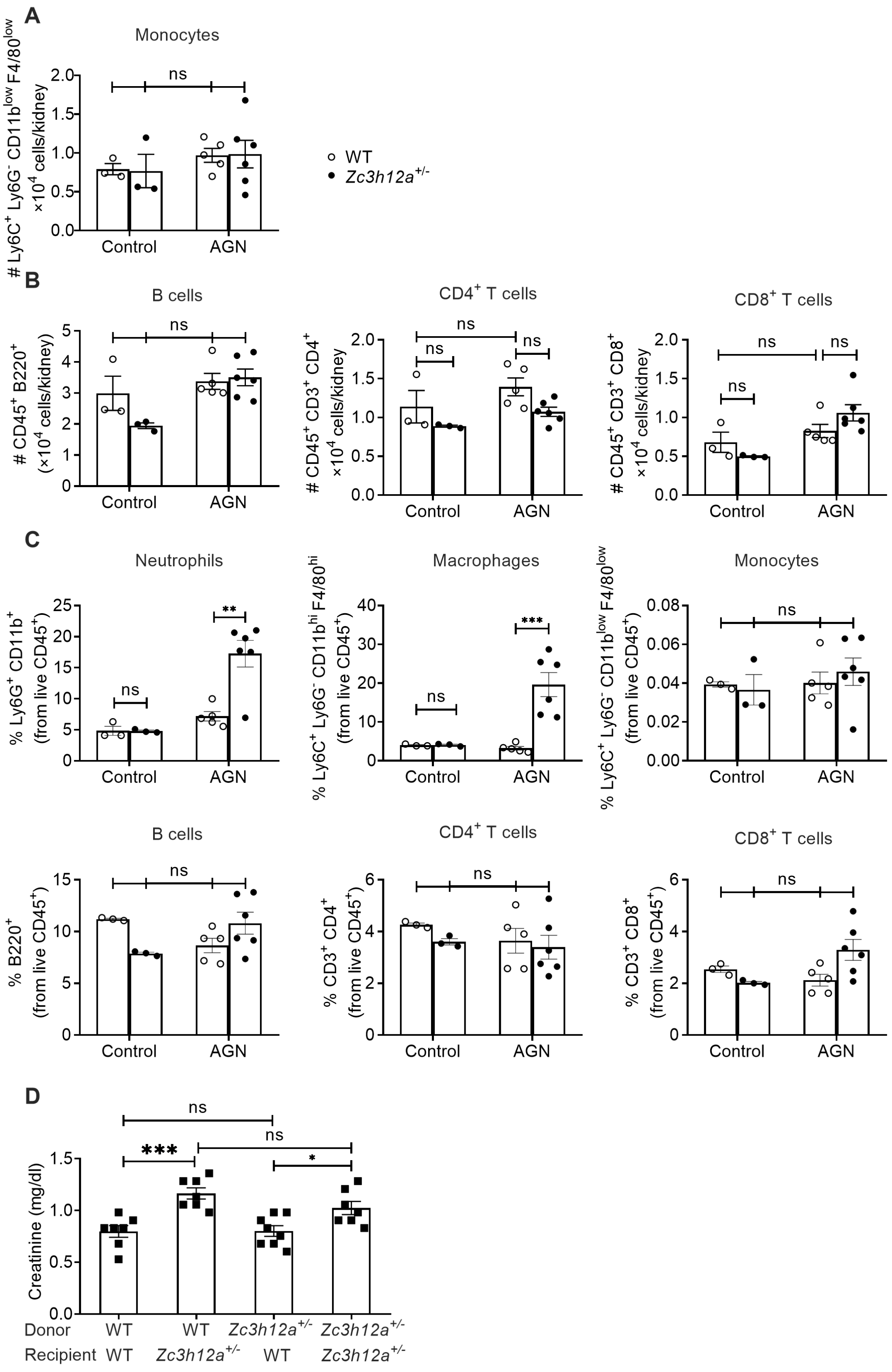

A
Monocytes
Ly6C+ Ly6G- CD11blow F4/80low ×10^4 cells/kidney
ns
WT
Zc3h12a+/-
Control
AGN
B
B cells
CD45+ B220+ (×10^4 cells/kidney)
CD4+ T cells
CD45+ CD3+ CD4+ ×10^4 cells/kidney
CD8+ T cells
CD45+ CD3+ CD8+ ×10^4 cells/kidney
C
Neutrophils
% Ly6G+ CD11b+ (from live CD45+)
Macrophages
% Ly6C+ Ly6G- CD11bhi F4/80hi (from live CD45+)
Monocytes
% Ly6C+ Ly6G- CD11blow F4/80low (from live CD45+)
B cells
% B220+ (from live CD45+)
CD4+ T cells
% CD3+ CD4+ (from live CD45+)
CD8+ T cells
% CD3+ CD8+ (from live CD45+)
**
***
D
Creatinine (mg/dl)
*
Donor WT WT Zc3h12a+/- Zc3h12a+/-
Recipient WT Zc3h12a+/- WT Zc3h12a+/-

Figure S4

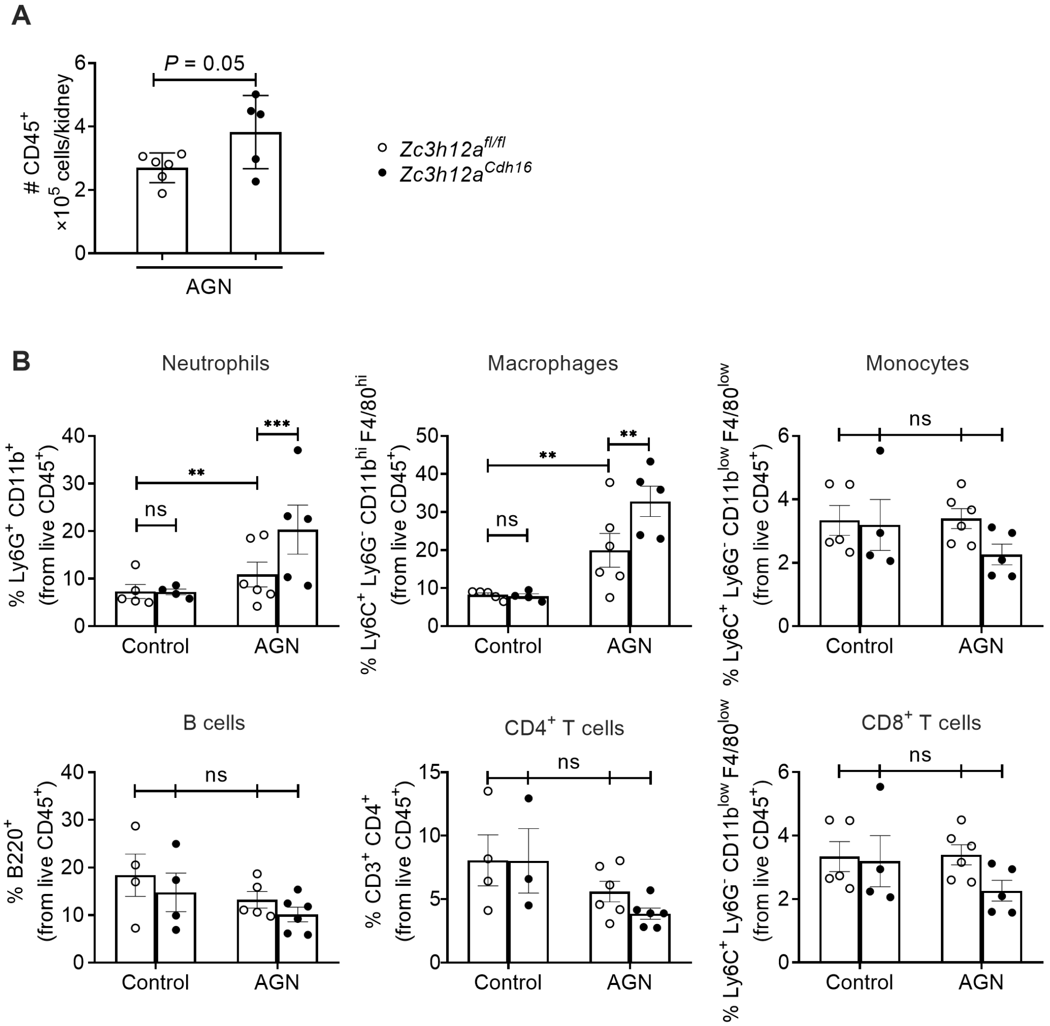

A
P = 0.05
# CD45+ ×10^5 cells/kidney
AGN
Zc3h12a fl/fl
Zc3h12a Cdh16
B
Neutrophils
% Ly6G+ CD11b+ (from live CD45+)
ns
**
***
Control
AGN
Macrophages
% Ly6C+ Ly6G- CD11b hi F4/80 hi (from live CD45+)
Monocytes
% Ly6C+ Ly6G- CD11b low F4/80 low (from live CD45+)
B cells
% B220+ (from live CD45+)
CD4+ T cells
% CD3+ CD4+ (from live CD45+)
CD8+ T cells

Figure S5

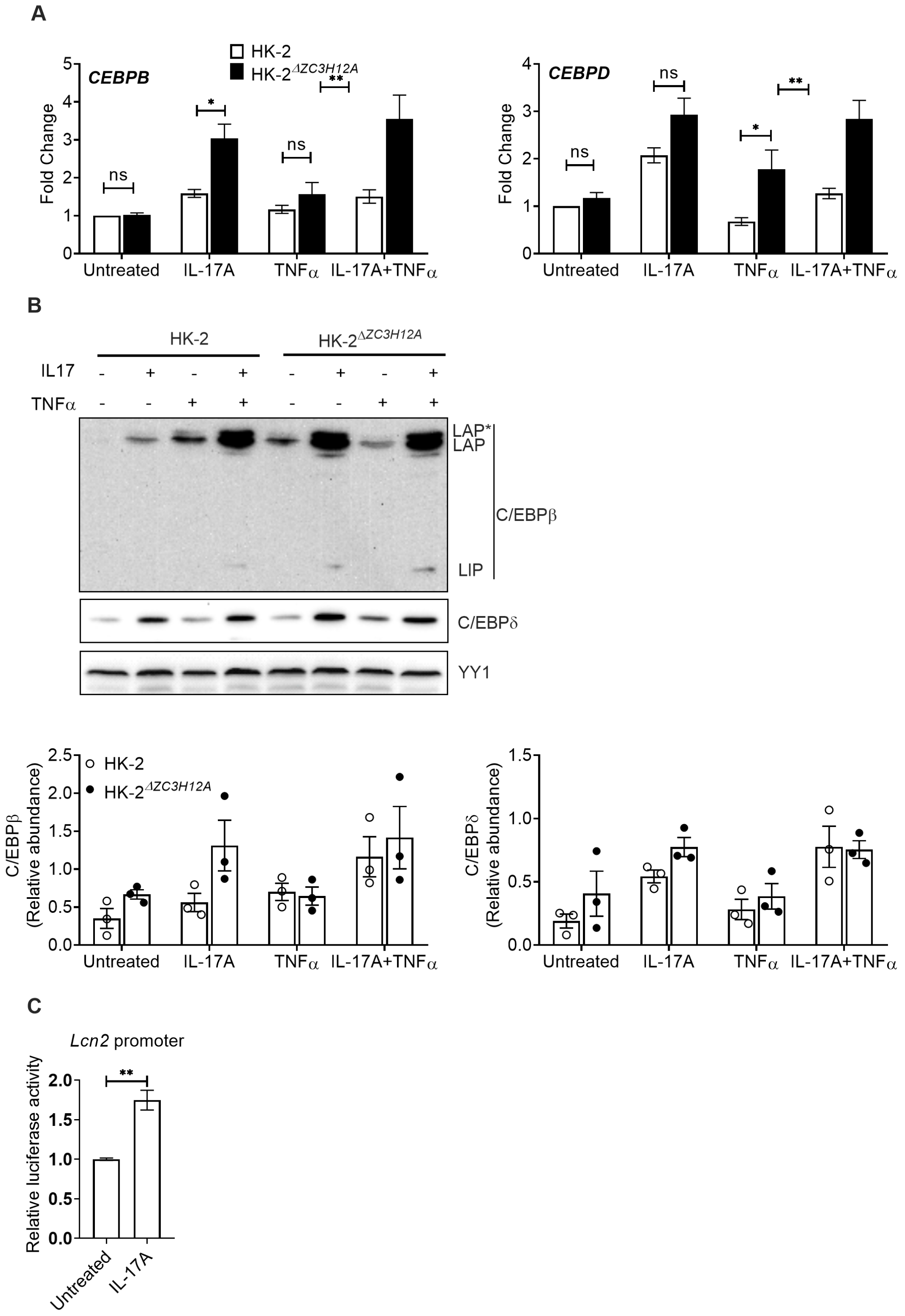
A
CEBPB
HK-2
HK-2^ΔZC3H12A
Fold Change
ns
*
**
Untreated
IL-17A
TNFα
IL-17A+TNFα
CEBPD
B
HK-2
HK-2^ΔZC3H12A
IL17
TNFα
LAP*
LAP
C/EBPβ
LIP
C/EBPδ
YY1
C/EBPβ (Relative abundance)
C/EBPδ (Relative abundance)
C
Lcn2 promoter
Relative luciferase activity
Untreated
IL-17A
